# A non-viral genome editing platform for site-specific insertion of large transgenes

**DOI:** 10.1186/s13287-020-01890-6

**Published:** 2020-09-03

**Authors:** Namrata Chaudhari, Amanda M. Rickard, Suki Roy, Peter Dröge, Harshyaa Makhija

**Affiliations:** 1 School of Biological Sciences, Nanyang Technological University, Singapore, 637551 Republic of Singapore; 2 Genea Biocells, 11099 North Torrey Pines Road, Suite 210, La Jolla, CA 92037 USA

**Keywords:** Genome engineering, Gene therapy, Embryonic stem cells, Large transgene, FVIII clotting protein, Lambda integrase, Site-specific tyrosine recombinase

## Abstract

**Background:**

The precise, functional and safe insertion of large DNA payloads into host genomes offers versatility in downstream genetic engineering-associated applications, spanning cell and gene therapies, therapeutic protein production, high-throughput cell-based drug screening and reporter cell lines amongst others. Employing viral- and non-viral-based genome engineering tools to achieve specific insertion of large DNA—despite being successful in *E. coli* and animal models—still pose challenges in the human system. In this study, we demonstrate the applicability of our lambda integrase-based genome insertion tool for human cell and gene therapy applications that require insertions of large functional genes, as exemplified by the integration of a functional copy of the *F8* gene and a Double Homeobox Protein 4 (DUX4)-based reporter cassette for potential hemophilia A gene therapy and facioscapulohumeral muscular dystrophy (FSHD)-based high-throughput drug screening purposes, respectively. Thus, we present a non-viral genome insertion tool for safe and functional delivery of large seamless DNA cargo into the human genome that can enable novel designer cell-based therapies.

**Methods:**

Previously, we have demonstrated the utility of our phage λ-integrase platform to generate seamless vectors and subsequently achieve functional integration of large-sized DNA payloads at defined loci in the human genome. To further explore this tool for therapeutic applications, we used pluripotent human embryonic stem cells (hESCs) to integrate large seamless vectors comprising a ‘gene of interest’. Clonal cell populations were screened for the correct integration events and further characterized by southern blotting, gene expression and protein activity assays. In the case of our hemophilia A-related study, clones were differentiated to confirm that the targeted locus is active after differentiation and actively express and secrete Factor VIII.

**Results:**

The two independent approaches demonstrated specific and functional insertions of a full-length blood clotting F8 expression cassette of ~ 10 kb and of a DUX4 reporter cassette of ~ 7 kb in hESCs.

**Conclusion:**

We present a versatile tool for site-specific human genome engineering with large transgenes for cell/gene therapies and other synthetic biology and biomedical applications.

## Background

Genetic insertions of large transgenes find utility in the design of gene therapies for monogenic diseases, innovative cell therapies, and in imparting multifunctionality to cells for biosynthetic applications [1]. A simple approach for the integration of large multi-transgene cassettes larger than 10 kb into the human genome remains a niche application domain where most of the tools (both viral- and non-viral-based) struggle to make an impact. This is due to problems of lack of specificity, undesirable genotoxicity, low efficiency and safety concerns. For example, adeno-associated viruses (AAVs) have a packaging limit of 4.7 kb, and within its capacity, it has shown promising clinical outcomes with long-term expression of truncated variants of *F8* (4371 bp) and *F9* (1257 bp) in hemophilia A and B patients, respectively. Although AAVs usually express transgenes as an episome, chromosomal integration still occurs either via homologous or non-homologous recombination pathways and can produce long-term effects [2, 3]. On the other hand, lentiviral-based vectors have superior payload capacity and carry inserts up to 18 kb; however, it is known that functional output and packaging efficiency significantly reduces as the load size increases > 8 kb [4–11]. Furthermore, viral-based transgenesis is cost and labour extensive and can lead to potential accentuating effects such as genotoxicity, oncogenicity and adverse humoral immune responses [12–15]. In contrast, non-viral CRISPR/Cas9 tools and other endonuclease-based genome editing (ZFNs and TALENs) systems are specific towards their target sequences, but their capability to routinely integrate payloads is somewhat limited to ~ 5 kb in size [16]. This is due to their inherent mechanistic principle of entirely relying on host-encoded recombination pathways such as homologous recombination that can be impaired in certain human cell types, especially in hES and somatic cells [17–22].

The most commonly used tool for large DNA transgenesis employs transposons that have been shown to integrate 8–10 kb DNA payloads [23]. However, their utility has been hindered by random transgene integration. To overcome these challenges, conventional genome engineering tools must be refined to successfully achieve functional insertion of large transgenes into the human genome. Several studies have employed combinatorial strategies of different editing tools to achieve specific insertion of large DNA [21]. Transposons are being explored in combination with CRISPR/Cas, called CRISPR-associated transposase system (CAST), to enable large DNA (~ 10 kb) integration at specific genomic locations and has, so far, only been validated in *E. coli* [24, 25]. However, another approach where piggyBac transposase was fused to catalytically inactive dCas9 demonstrated a successful ‘proof-of-concept’ in achieving the integration of the transgene at the CCR5 safe harbour site in HEK293 cells, thus enabling targeted delivery of large DNA cargos in the future [24, 26]. In addition, the CRISPR Cas systems have been paired with different homologous and non-homologous end joining (NHEJ) repair strategies to achieve large DNA knock-ins, an effective strategy in some eukaryotes but not in human systems [27–30]. Therefore, there is a void in the current genome editing toolbox to meet the need of functional large transgene insertions into the human genome safely at specific locations. Such an approach could substantially improve and enable downstream applications, spanning from engineered cell-based high-throughput drug screening, stem cells for regenerative medicine and cancer immunotherapies amongst others.

Important aspects of genome engineering include both integration of the desired DNA payload and disposing of undesired non-functional sequences, such as bacterial plasmid backbones that can elicit humoral responses due to immunogenic CpG motifs [31–37]. To achieve this, an alternative class of tools, site-specific recombinases (SSRs), are being employed to generate seamless vectors via intramolecular recombination using their respective recombination sites within the plasmid [38–40]. Thus, seamless vectors are circular supercoiled molecules obtained by eliminating the prokaryotic sequences that reduce the size of the vector by about 3 kb. This strategy can enable higher DNA uptake and reduced cell toxicity [41, 42]. In the context of human genome engineering, none of the SSRs tools has dual capability to produce and subsequently target specific endogenous sequences in the human genome. We previously reported a derivative of the phage lambda integrase (λ-Int) system which is proficient in targeting at endogenous Long INterspersed Elements (*LINE-1*) in the human genome with seamless vectors [43–45]. The derivative λ-Int system deploys self-sufficient intramolecular recombination to generate seamless vectors and executes specific human genome insertion by subsequent intermolecular recombination [44, 45]. Using this enhanced strategy, we also demonstrated specific targeting and sustained expression of CD19 chimeric antigen receptors (CARs) in hESCs for potential cancer immunotherapy-related applications [45].

The wild-type λ-Int system normally integrates the ~ 48 kb circular phage genome into the host genome. Here, we used the ability of our engineered λ-Int to perform large DNA insertions at specific genomic sites in human cells through our seamless vector approach, and exemplify the utility of our transgenesis tool for potential gene therapy approaches in hemophilia A and drug screening for FSHD disease. We demonstrate functional seamless transgenesis of both the ~ 10 kb full-length *F8* gene and a ~ 7 kb multi-reporter cassette into specific *LINE-1* sequences in hESCs. The demonstrated simplicity of our genome engineering tool provides the basis for broadly based economical applications in the future.

## Materials and methods

### Cell culture

The hESC line ‘Genea 019’ (Genea Biocells) was used in this study. The cells were cultured in BioCoat Collagen I-coated Plates (Corning) and maintained at 37 °C in 5% humidified CO_2_ and O_2_ atmosphere in M2 media (Genea Biocells). Media was supplied with serum and additionally supplemented with penicillin and streptomycin at 25 U/ml each (Gibco). Passaging solution and neutralization solution (Genea Biocells) were used for routine passaging of cells.

### Plasmids

To generate F8 expressing *pattP4X-pEF1a-FLF8-IRES-Neo-attH4X*, full-Length *F8* was amplified from *F8* expressing piggyBac vector (kindly provided by Prof. Akitsu Hotta, Kyoto University) using high-fidelity DNA polymerase and cloning primers 5.1F and 5.1R. The amplified F8 PCR product was cloned in the *AflII* linearized *pEF1a-IRES-Neo* vector (Plasmid #28019, Addgene) to generate *pEF1a-F8-IRES-Neo*. The *EF1a-F8-IRES-Neo* cassette was amplified using high fidelity DNA polymerase and cloning primers 7.1F and 7.1R and finally cloned into the master plasmid *pattP4X-attH4X* using *PstI*.

To generate *pattP4X-16BS-mNeon-PGKss-Puro-bpa-attH4X*, a linear fragment comprising of 16BS-mNeon flanked by *PstI* sites was synthesized (GenScript, USA) and cloned into the master plasmid using In-Fusion HD Cloning kit (Takara), eventually adding *16BS-mNeon* cassette in between *attP4X* and *attH4X* sequence. *PGKss-Puro* was then added to this plasmid by PCR amplification of the *PGKss-Puro-bpa* cassette from *pattP4X-PGKss-Puro-bpa-attH4X* (in-house), using the primers PGK_fwd_HR and Puro_bpa rev_HR. The PCR product was cloned into *pattP4X-16BS-mNeon-attH4X* using *NheI* as per the protocol of In-Fusion HD Cloning kit (Takara Bio USA), adding *PGKss-Puro-bpa* cassette downstream of *16BS-mNeon* cassette.

Cloning was performed using Q5 High Fidelity DNA Polymerase (New England Biolabs) and In-Fusion HD cloning kit (Takara). *E. coli* DH5α cells were used for transformation. Plasmids were extracted using QIAprep Spin miniprep kit (Qiagen) and EndoFree plasmid maxi kit (Qiagen).

### Generation of seamless vector via in vitro recombination using Int-h/218

The integrase-mediated in vitro recombination reaction for seamless vector generation was modified from the method described in [45]. Briefly, recombination was carried out in a reaction mixture (20 μl) containing 500 ng substrate vector, 10 mM TE buffer, pH 8.0, 150 mM KCl, 57 ng/μl of purified single chain Integration Host Factor (scIHF) [46] and partially purified Int-h/218 (33.25 ng/μl) [43, 47]. Sixty (30 μg DNA in total) reactions were incubated at 37 °C for 60 min and terminated by adding 0.5% SDS. Reactions were pooled and DNA was phenol/chloroform/isoamyl alcohol extracted and precipitated overnight using sodium acetate-ethanol. The reaction mixture containing unrecombined substrate plasmid and catenated circular DNA were digested with a suitable restriction enzyme (single cutter on the bacterial sequence of plasmid) and T5 exonuclease (NEB M0363) at 37 °C. The seamless vector was purified from the digestion mixture using phenol-chloroform extraction and ethanol precipitation of DNA.

### Transfection and antibiotic selection

Parental hESCs (250,000 cells/well) were seeded in 6-well plates overnight at 50% confluency. The following day, the cells were reverse co-transfected with the substrate or seamless vector along with Int-C3/Inactive Int expression plasmid using FuGENE HD Transfection Reagent (Promega) at a ratio of 1:3 (DNA: Reagent) using previously published protocol [44]. Forty-eight hours post-transfection, transfected cells were collected and replated onto 10 cm dishes. After 13–14 days of 300 ng/ml of puromycin or 100 μg/ml of neomycin (stock solution of 50 mg/ml in water, Gibco, Life Technologies) selection, surviving colonies were manually lifted, dissociated into single cells and reseeded for expansion initially in 96-well plates and later in 24-well plates.

### PCR screening to identify recombination events

Genomic DNA was isolated from parental hESCs and clones using the DNeasy Blood & Tissue Kit (Qiagen). Approximately 50 ng of genomic DNA from parental hESCs and clones was used as a template to amplify left and right recombination junctions. PCR was performed using GoTaq Flexi DNA polymerase (Promega) according to the manufacturer’s instructions. Primer sets were specific to vector and genomic DNA sequences adjacent to the site of integration. Primer positions and amplicon sizes are shown in figures (primer sequences are listed in Supplementary Table S1). PCR amplicons were gel extracted using QIAquick gel extraction kit (Qiagen) and examined by sequencing**.**

### Southern blot hybridization

Genomic DNA was isolated from parental hESCs and clones using the DNeasy Blood & Tissue Kit (Qiagen). Approximately 20 μg of each DNA was digested with a suitable restriction enzyme (New England Biolabs) overnight at 37 °C. Genomic DNA fragments were separated by electrophoresis on a 0.8% agarose gel in 1x TAE (Tris-Acetate-Boric acid) buffer, with 1 kb DNA marker ladder (New England Biolabs) and transferred onto a positively charged nylon membrane (GE Healthcare) via capillary transfer method. The DNA on the membrane was UV crosslinked and the membrane was probed at 48 °C with PCR-amplified DIG-labelled NeoR probe using the DIG-High Prime DNA Labelling and Detection Starter Kit II (Roche) as per the manufacturers’ protocol. The probe-target hybrids on the blot were detected by an AP-conjugated DIG-Antibody (Roche) using CSPD (Roche) as a substrate for chemiluminescence. The blots were exposed to X-Ray film (Kodak) and developed on a Kodak X-OMAT 2000 Processor.

### Gene expression

Total RNA from parental hESCs and clones was isolated using TRIzol reagent (Invitrogen). The RNA quality and quantity were assessed by Nanodrop UV-VIS spectrophotometer (Thermo Fisher Scientific). One microgram of total RNA from each sample was reverse transcribed to cDNA using the QuantiTect Reverse Transcription Kit (Qiagen). Using the QuantiNova SYBR Green PCR Kit (Qiagen), RT-qPCR was performed on the CFX96 Touch Real-Time PCR Detection System (Bio-Rad). The actin gene was amplified as an endogenous reference gene. Expression of the target gene was normalized to actin gene expression and represented as fold change using comparative CT method (2^-ΔΔ^CT method) [48].

### FVIII activity assays

Parental hESCs and clones were seeded in 96-well plates at ~ 70% confluence and culture supernatants were collected after 24 h. activity was determined by a fluorometric assay using the Factor VIIIa Activity Assay as per the manufacturer’s instructions. The assay was performed in a Corning 96-well microplate with a black flat bottom and the readings were recorded at kinetic mode (Ex/Em = 360/450 nm) using BioTek Cytation 5 cell imaging multimode reader for 8 h at 37 °C. The Factor VIII activity was normalized to cell viability and represented as fold change compared to parental hESCs.

### MTT assay

Cell viability was measured by MTT assay that quantifies the reduction of tetrazolium dye - MTT (3-[4,5-dimethyl thiazole-2-yl]-2,5-diphenyl tetrazolium bromide) in viable cells by mitochondrial NADPH-dependent cellular oxidoreductase enzymes [49]. MTT reagent (Sigma-Aldrich) was prepared at a concentration of 5 mg/ml in PBS. After collecting supernatants for Factor VIII activity, MTT reagent (10 μl) was added in wells (clones and parental hESCs) and incubated for 3 h at 37 °C. The medium in each well was replaced with DMSO to solubilize the purple-coloured formazan dye. The plate was mixed thoroughly and read for absorbance at 570 nm using BioTek Cytation 5 cell imaging multimode reader.

### Differentiation of hESCs

Parental hESCs and clones were differentiated with retinoic acid (RA; Sigma-Aldrich) over a period of 14 days as described previously [44]. Briefly, cells were initially cultured in DMEM containing 1 μM RA for 48 h and subsequently maintained in DMEM without RA for 12 days. Culture supernatants were used to measure Factor VIII activity and cells were collected for gene expression analysis.

### Statistical analysis

Statistical tests were performed using Graph Pad Prism6 software. Student’s unpaired *t* test was applied to compare between two groups. Data is represented as mean ± SEM and *p* value < 0.05 was considered statistically significant.

## Results

### Production of seamless *F8* targeting vector for site-specific transgenesis

We recently presented a phage λ integrase (Int)-mediated site-specific transgenesis platform capable of inserting large functional multi-transgene cassettes into a specific endogenous sequence, termed *att*H4x, within a subset of human *LINE-1* [44]. The *att*H4x sequence is present at about 900 locations throughout the human genome. An important improvement of our platform was the inclusion of supercoiled seamless target vectors devoid of prokaryotic DNA elements. This was achieved by using Int for in vitro/in vivo site-specific intramolecular recombination between two directly repeated recombination sequences (so-called attachment (*att*) sites) flanking the desired transgene expression cassette in a supercoiled parental substrate vector [44, 45]. Thus, besides eliminating unwanted bacterial sequences from the target vector, this approach also reduces the vector size and can enhance transfection efficiency, reduce innate immune responses and contribute to sustained gene expression in human cells [33, 50–52].

**Fig. 1:**
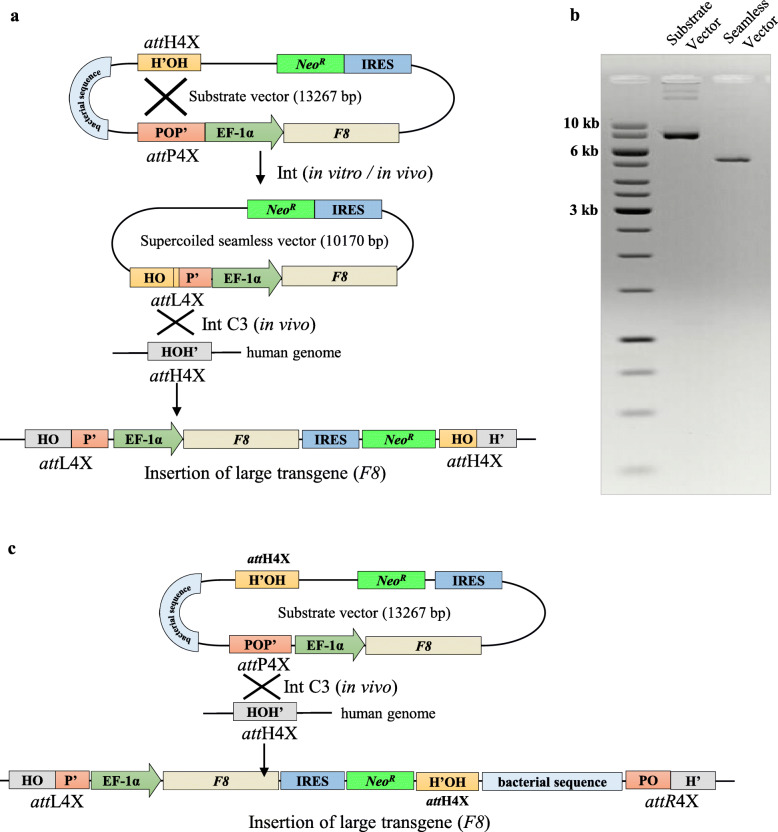
*F8* Seamless vector production and targeting strategy for genomic recombination of seamless vector with endogenous *att*H4x sites in *LINE-1*. **a** A pictorial representation of phage λ-mediated intramolecular in vitro/in vivo recombination between *att*H4X and *att*P4X (both present in the parental substrate vector) generating seamless vector *EF1α-F8-IRES-NeoR* with a recombinant *att*L4X junction, which can subsequently intracellularly recombine with *att*H4X (present in human genome *LINE-1).* Successful integration will form *att*L4X (left) and *att*H4X (right) recombinant sites flanking the cassette *EF1α-F8-IRES-NeoR* at the site of integration. **b** Agarose gel electrophoresis of parental substrate vector and *F8* seamless vector demonstrating their migration and quality. The supercoiled substrate vector (13,267 bp) migrates at ∼ 8 kb linear control DNA and supercoiled *F8* seamless vector (10,170 bp) migrates at ∼ 5.7 kb in a gel containing ethidium bromide. **c** A schematic representation of λ -mediated intracellular recombination of *att*P4X (present in the parental substrate vector) with *att*H4X (present in human genome *LINE-1).* Successful integration will form *att*L4X (left) and *att*R4X (right) recombinant sites flanking the cassette *EF1α-F8-IRES-NeoR* along with bacterial sequences at the site of integration

As a first step towards future autologous cell replacement therapies for hemophilia A, we employed this seamless vector transgenesis platform for site-specific integration of a functional, full-length *F8* expression cassette (10.1 kb) into the *att*H4X sequence in hESCs. The seamless target vector carries the *att*L4X recombination site and the EF1α promoter-driven *F8* gene expression cassette followed by an internal ribosome entry site (IRES)-driven neomycin resistance marker (NeoR). Targeted recombination into the genomic *att*H4X will generate *att*L4X and *att*H4X sequences flanking the inserted *F8* gene expression cassette (Fig. 1a). We used a modification of the previously published in vitro vector production protocol using purified Int [45] that now includes linearization of both the supercoiled bacterial backbone and remaining un-recombined substrate vector by restriction digest in conjunction with the degradation of linear and nicked DNA by phage T5 exonuclease. Simultaneous digestion of the in vitro recombination reaction products by restriction enzyme and T5 exonuclease greatly facilitated the production of sufficient amounts of highly purified supercoiled seamless *F8* vector (Fig. 1b).

### Targeted integration of *F8* seamless expression vectors

The in vitro manufactured seamless vector containing the *F8* expression cassette plus selection marker was co-introduced into hESCs together with Int expression vector to establish *F8* knock-in clones. Importantly, since the intramolecular recombination reaction on the substrate vector can also occur inside cells before intermolecular recombination with the genome (Fig. 1a), we also tested this alternate route of integration and introduced the unrecombined substrate vector to determine whether in vitro seamless vector production can be bypassed by intramolecular recombination inside the cell. In parallel, this would also explore the possibility of insertion the entire substrate vector into genomic *att*H4X via recombination with *att*P4X (Fig. 1c).

**Fig. 2:**
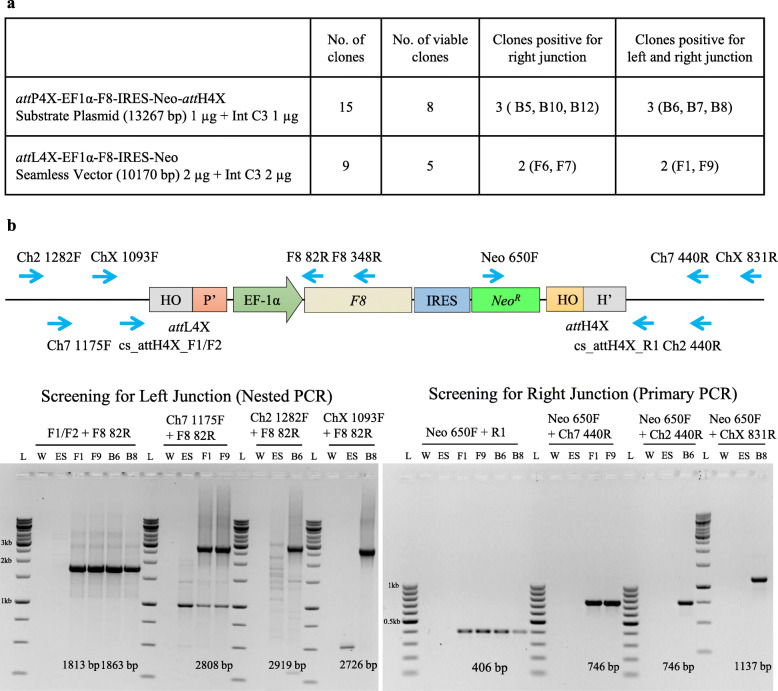
PCR analysis of left and right recombination junction and characterization of site-specific integration of seamless vector at *LINE-1* in clones. **a** The table includes the amount and combinations of vector (substrate vector and seamless vector) and Integrase expression vectors transfected in hESCs to establish neomycin resistant *F8* knock in clones. **b** Schematics of left and right junction of the integrated seamless vector in *LINE-1.* Gel in left panel: PCR analysis showing products of semi-nested PCR obtained with forward primers specific to *LINE-1* (F1/F2)/genomic locus (Ch7 1175F, Ch2 1282F, ChX 1093F) and reverse primer (82R) in *F8* using template from primary PCR performed using same forward primers and reverse primer (348R) in *F8.* Gel in right panel: PCR analysis showing products of PCR obtained with forward primer in *NeoR* gene (Neo 650 F) and reverse primers specific to *LINE-1* (R1)/genomic locus (Ch7 440R, Ch2 440R, ChX 831R). Arrows indicate primer position and orientation. Expected PCR amplicon sizes are mentioned for each primer pair at the bottom of each gel. Lanes: L, 1 kb DNA ladder; W, no DNA control; ES, genomic DNA from parental hESCs; F1, F9, B6, B8, genomic DNA from clones. 50 ng of template DNA was used for primary PCRs and 1 μl of primary PCR reaction was used as template for semi-nested PCRs

Substrate and seamless vectors were co-transfected in hESCs with either an expression vector for variant Int-C3 or a catalytically inactive integrase Int INA [45]. Two days after co-transfection, G418 selection was applied resulting in stable cell clones after 15 days. Importantly, transfection with Int INA resulted in 50% fewer clones compared to Int-C3. A total of fifteen and nine hESC clones were obtained by co-transfection of catalytically active Int-C3 with the substrate and seamless vector, respectively (Fig. 2a). Viable clones were expanded, and genomic DNA was subjected to junction PCR analysis using consensus genomic primers (cs_*att*H4X_F1/F2 and cs_*att*H4X_R1) designed to bind adjacent to *att*H4X sites within the corresponding *LINE-1* (Fig. 2b) [44, 45]. Accordingly, successful integration of the *F8* expression cassette in any of the *LINE-1* loci will result in PCR amplicons specific for left and right recombinant junctions using combinations of the genomic (*LINE-1*) and cassette-specific primers in *F8* or *NeoR* (Fig. 2b).

Co-transfection with substrate vector and Int-C3 can convert the episomal substrate vector into a seamless vector via intramolecular recombination. Hence, either the entire substrate (via *att*P4X) or the smaller seamless vector (via *att*L4X) can recombine with the genomic *att*H4X sequence (Fig. 1a, c). Analyzing the respective outcome after co-transfection of the entire substrate vector with Int-C3 expression vector by PCR would result in the same product for the left recombination junction but yields two distinct products for the right junction PCR, thus allowing us to distinguish between the two scenarios. PCR screening for substrate vector transfections revealed only integration events of the seamless vector via *att*L4X and genomic recombination. We found that three out of eight clones (B6, B7 and B8) were positive for PCR analysis of both junctions (Fig. 2b) indicating that Int-C3 had first intramolecularly recombined the transfected substrate vector and subsequently integrated the seamless vector into the genomic *att*H4X of *LINE-1*.

Transfection with in vitro generated seamless vector resulted in four out of nine viable clones that were positive for right junction PCR; two clones (F1 and F9) were tested positive for both junctions. As shown in Fig. 2b, semi-nested PCRs were performed in order to obtain sufficient products from all left junctions for sequencing, whereas right junction PCR amplicons were identified in primary PCRs (Fig. 2b). PCR products obtained using *LINE-1*-specific primers were subjected to sequence analysis to identify the genomic locus of *F8* cassette integration. The corresponding targeted *LINE-1* loci were subsequently verified by PCR/sequencing using chromosome-specific primers (Fig. 2b, right panel). Our combined results demonstrate that at least five clones (B6, B7, B8, F1, F9) harboured the complete *F8* expression cassette and that three different *LINE-1* loci were targeted by Int-mediated recombination (Supplementary Table 2).

### Single copy *F8* seamless vector insertion at endogenous *att*H4X sites

**Fig. 3:**
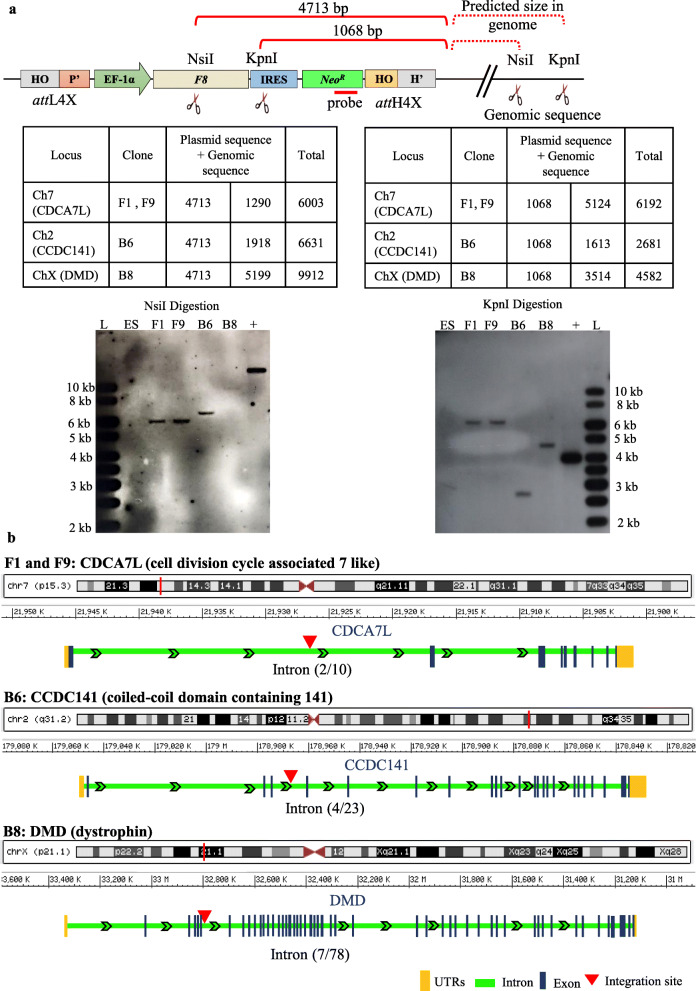
Southern blot hybridization of clones targeted with *F8* seamless vector. **a** Schematics of integrated *F8* seamless vector at the *LINE-1* with information on location of restriction sites within the cassette and in the hESC genome. The Table summarizes the targeted locus and genomic location of seamless vector integration for the clones, based on the genomic fragment sizes. Total genomic DNA from parental hESCs and clones harbouring the complete *F8* seamless vector was digested with *NsiI* and *KpnI* and subjected to hybridization with DIG-labelled PCR probe complementary to 309 bp in *NeoR* gene. Bands indicate *NeoR* gene containing genomic fragments which correlate with the predicted size thereby confirming single copy *F8* seamless vector integration at *LINE-1*. L, 1 kb DNA ladder; ES, genomic DNA from parental hESCs; F1, F9, B6, B8, genomic DNA from clones; + in NsiI Digestion indicates 0.1 ng of linearized substrate vector; + in KpnI Digestion indicates 0.1 ng of *NeoR* containing KpnI digested fragment (3969 bp) of substrate vector. **b** An illustration of the location of transgene integration in chromosomes for the targeted clones

We employed Southern blot hybridization to confirm seamless vector insertions at the identified loci and, furthermore, to determine if only a single copy of the *F8* expression cassette has been site-specifically integrated into the *LINE-1*. Two restriction endonucleases with recognition sites within the cassette and in the vicinity of the three predicted targeted *LINE-1* loci were independently used for digestion of genomic DNA. Using a vector-internal probe hybridizing to *NeoR*, it was possible to identify single-copy insertions at the three loci based on restriction fragment patterns (Fig. 3a).

The Southern blots obtained with *Nsi*I and *Kpn*I-digested genomic DNA from four out of the five above-mentioned clones, and untargeted hESCs DNA as control, clearly revealed single-copy integration of the seamless cassette for each clone/locus (Fig. 3a) and confirmed the stable integration of the seamless vector in intron 2 of *CDCA7L* (Cell Division Cycle Associated b 7 Like; Chr7) in clones F1 and F9, intron 4 of *CCDC141* (Coiled-Coil Domain Containing 141, Chr2) in clone B6 and intron 7 of *DMD* (Duchenne Muscular Dystrophy, ChrX) in clone B8 (Fig. 3a, b, Supplementary Table 2). With respect to clone B7, the Southern blot data suggested the existence of restriction site polymorphism near the targeted *LINE-1* locus (data not shown) and hence was not analysed further. Altogether these findings demonstrate the ability of our transgenesis tool to target endogenous *att*H4X sites within *LINE-1* elements with a 10.1-kb-sized therapeutic gene expression cassette. As exemplified by the independent targeting of the CDCA7L locus on chromosome 7 (for clones F1 and F9), the data also revealed the possible existence of hot-spot recombination loci for targeted transgene insertion mediated by mutant phage lambda Int-C3 [45].

### *F8* expression and catalytic FVIII activity in *LINE-1* targeted clones

**Fig. 4:**
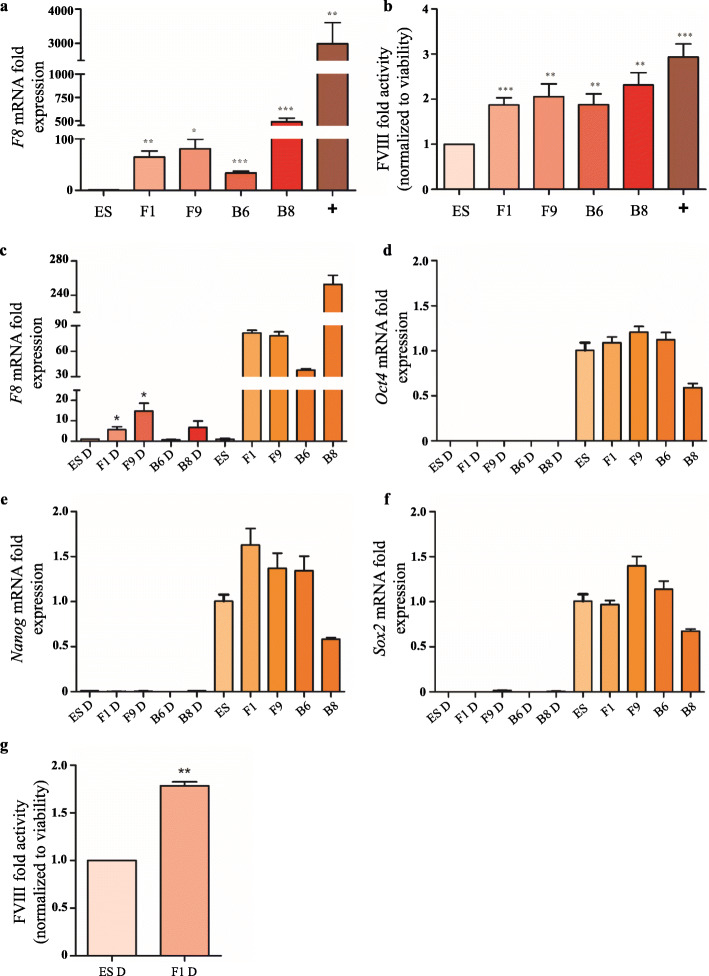
Gene expression and FVIII activity in hESCs and transgenic clones. **a** *F8* gene expression was determined by RT-qPCR analysis and performed at 24 h for *F8* mRNA expression in parental hESCs cells, transgenic clones and transiently substrate vector-transfected hESCs. *F8* mRNA expression was normalized to the level of invariant control human beta-actin and represented as fold change compared to parental hESCs. ES, cDNA from parental hESCs; F1, F9, B6, B8, cDNA from transgenic clones; + indicates transiently transfected hESCs with 1 μg of substrate vector. **b** FVIII activity in hESCs and transgenic clones. 48 h culture supernatants of parental hESCs cells, clones and transiently transfected hESCs were subjected to FVIII fluorometric activity assay to measure the secreted FVIII. The FVIII fold activity was normalized to cell viability and represented as fold change compared to values obtained with parental hESCs. Cell viability was measured using the MTT assay. ES, parental hESCs; F1, F9, B6, B8, clones; + indicates transiently transfected hESCs with 100 ng of substrate vector. **c–f** Gene expression in retinoic acid differentiated hESCs and clones. The RT-qPCR analysis was performed for *F8* and pluripotency markers *Oct4*, *Nanog*, *Sox2* mRNA expression in differentiated parental hESCs cells and transgenic clones on day 14 of differentiation. Corresponding gene expression in differentiated hESCs/clones was compared to that in undifferentiated hESCs/clones. mRNA expression was normalized to the level of invariant control human beta-actin and represented as fold change compared to respective parental/differentiated hESCs. **g** FVIII activity in differentiated hESCs and transgenic clone F1. Culture supernatants of differentiated hESCs and clone F1 were subjected to FVIII fluorometric activity assay to measure the secreted FVIII. The FVIII fold activity is represented as fold change compared to differentiated parental hESCs. ES, parental hESCs; F1, F9, B6, B8, transgenic clones; D denotes retinoic acid differentiated hESCs/clones

We next investigated if the targeted loci permitted sustained transgene expression. Quantitative RT-PCR analysis was performed to analyse the *F8* mRNA expression levels of the four *F8* transgenic clones (F1, F9, B6 and B8) normalized to the endogenous *F8* levels in untargeted hESCs. We observed a significant increase in the amount of *F8* mRNA in all transgenic clones (Fig. 4a). We included untargeted hESCs transiently transfected with the substrate F8 expression vector (1 μg) as a positive control, which, expectedly, showed the highest expression levels (Fig. 4a). These data demonstrated that the *EF1α-F8-IRES-NeoR* expression cassette is sustainably expressed in hESCs from these three targeted *LINE-1* loci.

We also determined if the produced *F8* mRNA was translated into protein and secreted from hESCs into the media in a biologically active form. We examined FVIII activity by a fluorometric assay in hESC culture supernatants, using again transiently transfected (100 ng) hESCs as positive and parental hESCs as negative controls. The fluorometric assay measures the ability of activated FVIIIa to generate Factor Xa in the presence of calcium and phospholipids, which further proteolytically cleaves a specific substrate to release a fluorophore that can be quantified. The FVIII activity was normalized to untargeted hESCs and to cell viability as measured by MTT assays to account for possible differences in cell density and growth rates of clones. Coinciding with the observed increase in *F8* mRNA expression, we found a significant increase in FVIII activity with all targeted hESCs clones and transiently transfected cells (Fig. 4b). Interestingly, we also noted that untargeted hESCs did express a substantial level of biologically active FVIII protein when compared with unexposed cell culture media as negative control, which may open interesting possibilities for non-recombinant FVIII production at a larger scale using hESC fermenters. Taken together, these results clearly indicated that the *LINE-1*-targeted cell clones, regardless of the transgene locus, produced biologically active FVIII and that clone B8 exhibited both the highest *F8* mRNA expression and protein activity.

Since many future applications of hESCs and induced pluripotent stem cells (iPSCs) will likely involve differentiation of stem cells into specific desired cell types, e.g. platelets, we next tested how *F8* transgene expression might be affected by the differentiation status of our targeted hESC clones. Hence, we employed an established retinoic acid (RA)-induced differentiation protocol which typically results in a mixture of various cell lineages and differentiation states when hESCs are cultured in DMEM containing 1 μM RA for 48 h and subsequently maintained in DMEM w/o RA for 12 days [53]. The results showed that the expression of the *F8* transgene cassette in the four differentiated cell clones was substantially reduced when compared to undifferentiated hESCs, but remained significantly higher in the two clones that carry the transgene in the same genomic locus (clones F1 and F9) compared to the endogenous *F8* transcript levels in parental differentiated cells (Fig. 4c). Control qRT-PCRs measuring expression of the key pluripotency factor genes *Oct4*, *Nanog* and *Sox2* confirmed that the most cells in the transgenic hESC clones and parental hESCs had lost their pluripotent stem cell state (Fig. 4d–f). Furthermore, FVIII activity tests revealed that differentiated cells from clone F1 are still secreting biologically active clotting factor when compared to differentiated untargeted cells (Fig. 4g).

### λ-Int-mediated reporter insertion for drug screening applications in FSHD disease

**Fig. 5:**
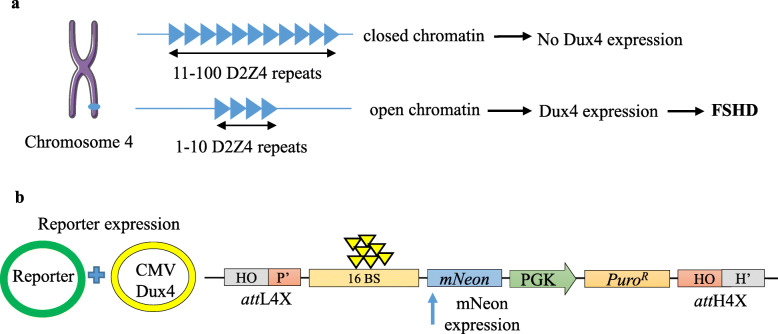
Schematic representation of disease modelling for FSHD, and proposed methodology for potential drug screening. **a** The genetic defect in the *DUX4* gene present in the repeats of the macro-satellite array (D4Z4) at chromosome 4q35 leads to array contraction to < 11 repeats and chromatin relaxation causing aberrant expression of the transcription factor DUX4 causing FSHD disease. **b** An illustration of transiently testing our reporter construct

**Fig. 6:**
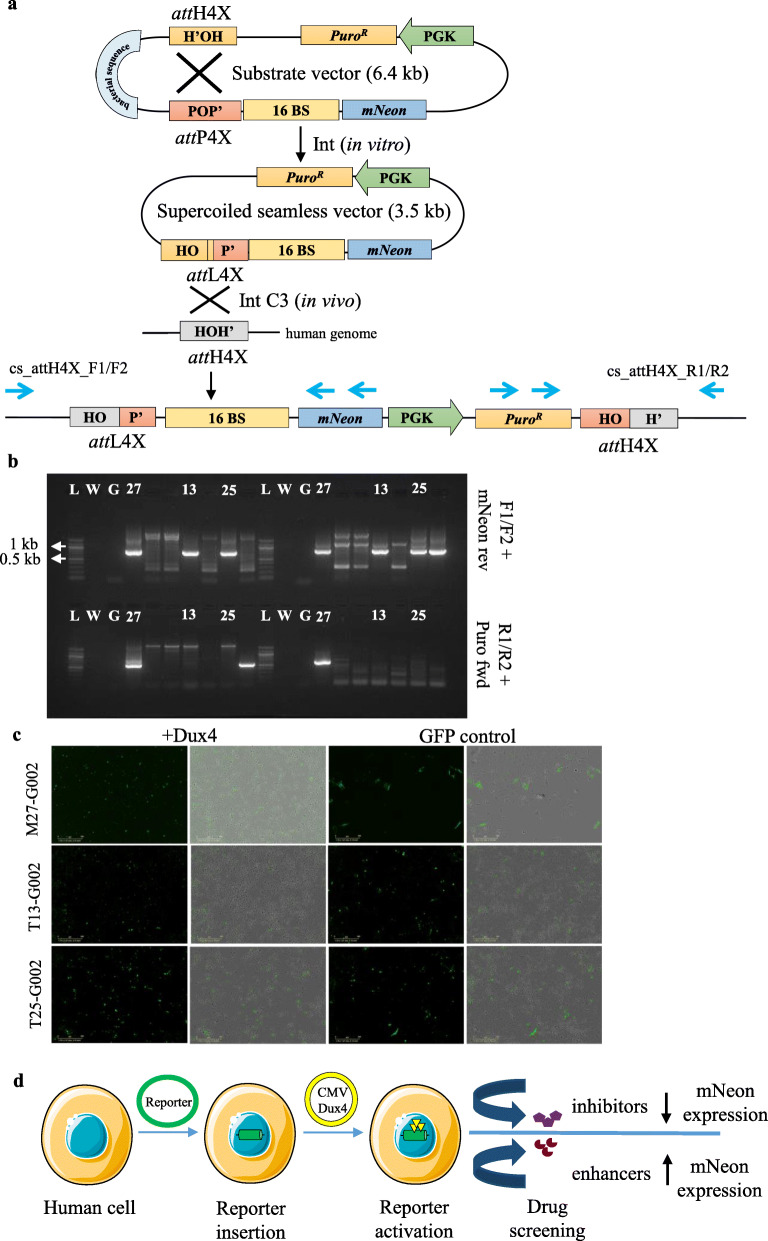
Seamless vector production and targeting strategy for *DUX4-mNeon* reporter cassette at endogenous *att*H4x sites in *LINE-1*. **a** A schematic representation of λ-Int mediated in vitro intramolecular recombination between *att*H4X and *att*P4X (both present in the parental substrate vector) generating DUX4-mNeon reporter seamless vector with a recombinant *att*L4X junction, which can subsequently intracellularly recombine with *att*H4X*.* Successful integration of the reporter resulted in *att*L4X (left) and *att*H4X (right) recombinant sites flanking the site of integration. **b** PCR analysis using genomic DNA from the puromycin resistant clones (obtained with co-transfection of DUX4-mNeon reporter seamless vector and Int expressing vector Int-C3) resulted in three clones (M27, T13, T25) positive for left junction with forward primers specific to *LINE-1* (F1/F2) and reverse primer (mNeon rev) and a M27 clone positive for right junction with reverse primers specific to *LINE-1* (R1/R2) and forward primer (Puro fwd). Lanes: L, 1 kb DNA ladder; W, no DNA control; G, genomic DNA from parental hESCs; 27,13,25; genomic DNA from M27, T13 and T25 puromycin resistant clones. **c** Transfection of transgenic clones M27, T13 andT25 with DUX4 expression vector *pCMV-DUX4* triggered mNeon expression in a substantial fraction of cells at day 2, indicating the functionality of the Dux4 binding sites of the integrated reporter. GFP-control, vector *pCMV-GFP* was used as a transfection control. **d** An illustration of the future application of our proposed methodology for high-throughput drug screening upon mNeon reporter activation with CMV-DUX4 plasmid. The reporter activity (mNeon expression) can be modulated depending on the compounds (inhibitors/activators) used for the screening

The human *DUX4* gene is located within a D4Z4 sequence repeat array in the subtelomeric region of chromosome 4q35. It is known that contraction of these D4Z4 macro-satellite sequences is associated with decreased cytosine methylation and an open chromatin structure, leading to infrequent sporadic expression of the *DUX4* gene in the skeletal muscle that results in facioscapulohumeral muscular dystrophy (FSHD) [54–56] (Fig. 5a). Given that DUX4 expression is difficult to detect in FSHD muscle cells, we employed our transgenesis system to generate a seamless vector comprising of a cassette harbouring a DUX4-responsive artificial promoter with 16 DUX4 binding sites upstream of a reporter gene (mNeon/fluorescent protein) and a downstream antibiotic selection cassette (PuroR driven by the PGK promoter: Fig. 5b). The mNeon expression as a readout was first validated with the episomal reporter by co-transfecting a DUX4 protein-expressing construct (*pCMV-DUX4*) into hESCs (Fig. 5b and data not shown). In order to generate the stable DUX4 reporter cell lines, our transgenesis platform was used to integrate the seamless reporter vector into *LINE-1* of hESCs (Fig. 6a). PCR analysis confirmed both left and right junctions indicating specific and complete integration of the reporter cassette in three transgenic cell clones (M27, T13, T25) (Fig. 6b, Supplementary Table 2). The functionality of the inserted reporter in these clones was confirmed by ectopic expression of the DUX4 protein using *pCMV-DUX4* expression vector.

As shown by fluorescence microcopy, activation of reporter expression in the three clonal cell lines can be achieved via transient expression of DUX4 (Fig. 6c). Importantly, the transfection efficiency in these reporter cell lines is sufficient to transiently express DUX4 and activate the reporter in a sufficient number of cells  for downstream applications. For example, potential high-throughput small compound screening can be performed on DUX4-activated cells (within a 24–48 h time window) to identify molecules that antagonize DUX4-mediated activation of the mNeon reporter (Fig. 6d) and thereby identify potential lead compounds.

## Discussion

Genetic engineering attributes that offer flexibility for large transgene insertions equivalent to 10 kb or more can have profound implications for cell/gene therapy and synthetic biology applications. However, as the genomic transgene insert size increases, multiple genotoxic effects due to random integrations, epigenetic silencing and chromosomal aberrations, amongst others, represent potential complications. Therefore, both versatility and safety features of genome editing tools are critical, especially for gene therapy applications of monogenic diseases that necessitate large transgene insertions for curative outcomes. Hemophilia A (*F8* coding sequence − 7 kb), DMD (*Dystrophin* coding sequence − 14 kb) and skin disease Recessive Dystrophic Epidermolysis Bullosa (*COL7A1* coding sequence − 9 kb) are examples of diseases for which replacement corrections of dysfunctional large genes could yield clinical benefits. In order to validate the utility of our previously reported λ-Int-based seamless transgenesis tool [44, 45] in achieving large DNA transgenesis, we have demonstrated here its use in the insertion of the full-length *F8* gene for hemophilia A as an example of a disease model.

Gene therapies for hemophilia A provides a tractable alternative to the present standard of care confined to prophylaxis, management of bleeding incidences and replacement therapy that includes repeated infusion of clotting factors to replace the missing/low endogenous FVIII protein [57–62]. Ideally, replacing the dysfunctional *F8* gene with a functional copy would be the most desirable way to benefit more than 400,000 affected hemophilia A patients worldwide [63, 64], but such genome engineering pursuits are extremely challenging owing to the large size of the gene [64, 65]. Hence, truncated *F8* variants as a substitute have been pursued to mimic FVIII-mediated physiological coagulation effects. AAV and other vectors have been widely used as a carrier for the truncated version of the *F8* gene; however, certain safety issues persist [64, 66–68]. An example of remaining adverse virus-mediated oncogenic effects has been concretely pointed in a canine model of hemophilia administered with AAV gene therapy in a decade long follow-up study, wherein DNA payload insertion was evidenced near genes that regulate cell growth [69, 70]. Many precedented ex vivo pioneering studies [71–76] have also been attempted to either genetically correct or introduce a separate functional copy of truncated *F8* into different types of cells by lentiviral, transposons and CRISPR Cas systems with a fair degree of success, yet still requiring significant improvements. In addition, lentivirus-based transduction of truncated *F8* variants into patient-derived iPSCs and directed differentiation to megakaryocyte [75] and endothelial cell-lineage [74] for functional FVIII production have achieved some success, albeit some adverse effects of random integrations linger. CRISPR Cas tools were also used to correct *F8* chromosomal inversions in patient-derived iPSCs and subsequent liver endothelial differentiation, an approach that could only benefit a subset of hemophilia patients who harbour such inversions [71]. Contrastingly, a CRISPR-Cas-mediated universal gene-correction knock-in strategy of introducing BDD-F8 gene at the endogenous F8 locus of hemophilia A patient-derived iPSCs differentiated into endothelial cells also did not yield optimal levels of FVIII [76]. This could be because the human *F8* locus is located on the X-chromosome and only one copy has been inserted at this locus which did not allow sufficient expression and yield of the FVIII protein. In addition, deletion of the protein’s B-domain results in a reduced rate of FVIII secretion, which could be attributed to misfolding and degradation of the BDD-FVIII protein compared to the full-length FVIII protein. Furthermore, this approach is marred with common issues of CRISPR, including indels, chromosomal aberrations and translocations [76]. A plausible direction of genome-editing strategies may involve introducing the *F8* coding sequence into putative safe harbour and high expression loci, such as AAVS1 or CCR5, but such approaches need to be rigorously evaluated. To this end, non-viral tools like transcription activator-like effector nickases (TALENickases) identified the multicopy ribosomal DNA (rDNA) locus as a safe and effective target for *F8* gene integrations and expression in hemophilia A-affected iPSCs. Unfortunately, they achieved a significant increase in the FVIII protein in the lysates of the targeted iPSCs but failed to achieve desirable FVIII protein in cell supernatants, indicating potential problems with folding and secretion of the FVIII protein [72].

To address the complex issues with hemophilia A gene therapy designs, we conceived a non-viral-based transgenesis of *F8* at potentially safe harbour sites in human ESC genome. We took advantage of our previously reported λ-Int system to generate seamless vectors harbouring the full-length *F8* gene using in vitro site-specific intramolecular recombination between two DNA recombination sequences (*att*H4X and *att*P4X) [44, 45] flanking the *F8* expression cassette in a 14-kb supercoiled parental substrate plasmid. Our seamless vector approach should minimize potential adverse host immune responses to bacterial sequences [31–37]. The *att*L4x harbouring ~ 10.1 kb *F8* seamless expression vector is then targeted to *att*H4x in the hESC genome. This approach also reduces the vector size, which, in turn, enhances DNA transfer. Our transgenesis strategy is potentially superior to Piggy Bac transposon-mediated full-length *F8* insertion with respect to controlled and specific transgene insertion at predetermined *LINE-1* sites [77]. The Piggy Bac system offers no control over integration sites, which bears a potential risk for insertional mutagenesis and unwanted genotoxicities [77–79]. A paralleled approach in our study of introducing the substrate plasmid for Int-C3 to catalyze intracellular intramolecular recombination to convert the episomal substrate vector into a seamless vector before integration into *LINE-1* elements is an important advance since it greatly simplifies the entire platform by eliminating seamless vector production at a larger scale in vitro. However, further experiments need to verify that the circular bacterial backbone DNA that is generated by intramolecular recombination inside cells is not randomly inserted in the host cells’ genome.

Our proof-of-concept study with transgenesis of *F8* resulted in five hESC clones (B6, B7, B8, F1, F9) that harboured the complete *F8* expression cassette in three different *LINE-1* loci. Southern blot and sequencing analysis confirmed stable single copy integrations at so-called *LINE-1* hot spots in four clones, a feature that will further simplify our platform technology and can be exploited in the future with other transgene constructs. Interestingly, the targeting site in clone F1 is identical to hotspots documented in our previous report [45]. This locus lies on chromosome 7 and is part of an intron 2 of *CDCA7L* responsible for regulation of cell division and apoptosis signalling pathway. We confirmed the expression and activity of the *F8* transgene from this targeted locus. We also showed that *F8* transgene expression can be retained in differentiated hESCs, an important validation for our technology’s use in future stem cell and cell therapy approaches. The fact that we can target several endogenous *att*H4X sequences in parallel and test for functional transgene expression in differentiated cells represents an additional bonus of our transgenesis method to eventually generate the desired transgenic cell product.

In a second approach, we expanded the applicability of our platform for the further development of reporter cell lines for drug screening applications. We had previously generated a hESC-derived pluripotency reporter cell line that has already been successfully used in safety assessments of lead compounds for the treatment of tuberculosis [44, 80]. Here, we employed a seamless transgenesis approach for hESC-derived reporter cells related to FSHD disease. FSHD is a genetic muscle disorder caused by the loss of transcriptional repression of *DUX4* gene, resulting in its aberrant expression and subsequent progressive muscle wasting predominantly in the face, shoulder blades and upper arms [81, 82]. The DUX4 protein is a transcription factor that targets a large set of genes and initiates a cascade of downstream signalling pathways that inhibit myogenesis and induces oxidative stress and cell death in FSHD skeletal muscle [83–85]. Various efforts are underway to model the disease in cultured cells for further studies of FSHD and to identify molecules that would interfere with pathogenic DUX4 expression or activity [84–87]. Given the high transfection efficiency that we can achieve with hES cells and their ability to differentiate into muscle lineage, herein, we reported the development of an alternative hESC-based reporter system comprised of large gene(s) cassette that can be adapted for high-throughput screening of drugs for FSHD disease. We constructed a DUX4 target gene reporter comprising of binding sites of DUX4 driving the *mNeon* gene that responds to DUX4 stimulation. We demonstrated that ectopic expression of DUX4 protein triggered the expression of the fluorescent reporter. We think it is feasible that these cell lines can be employed for high-throughput drug screening to identify small lead compounds that suppress DUX4’s activity as a transcriptional activator.

## Conclusion

We presented a simple λ-Int transgenesis platform as a non-viral alternative to achieve large transgenic insertions into the human genome for cell/gene therapy and synthetic biology applications, including drug screening.

## Supplementary information

**Additional file 1.**

## Data Availability

All data generated during this study are included in this published article and its supplementary information file. Research findings are available from the corresponding author upon reasonable request.

## Abbreviations

hESCs: Human Embryonic Stem Cells
DUX4: Double Homeobox Protein 4
FSHD: Facioscapulohumeral muscular dystrophy
CAST: CRISPR-associated transposase system
NHEJ: Non-homologous end joining
SSRs: Site-specific recombinases
*LINE-1*: Long INterspersed Elements
CARs: Chimeric Antigen Receptors
scIHF: single chain Integration Host Factor
IRES: Internal Ribosome Entry Site
*CDCA7L*: Cell Division Cycle Associated- 7 Like
*CCDC141*: Coiled-Coil Domain Containing 141
*DMD*: Duchenne Muscular Dystrophy
