# Supplementary material for: A non-viral genome editing platform for site-specific insertion of large transgenes
Source: Stem Cell Res Ther. 2020 Sep 3;11:380. doi: 10.1186/s13287-020-01890-6 (PMC7650303; doi:10.1186/s13287-020-01890-6)
Supplement: Supplementary file 1 — Additional file 1. [file 13287_2020_1890_MOESM1_ESM.pdf]

SUPPLEMENTARY DATA

Supplementary Table 1

List of Primers (5’-3’)

Human β actin F	AGAGCTACGAGCTGCCTGAC
Human β actin R	AGCACTGTGTTGGCGTACAG
F8 RT F2	AAGACTCACATTGATGGCCCATC
F8 RT R2	CTGTCATGAATCAAAGGTGTCAC
Neo_Fwd probe	GCTCCTGCCGAGAAAGTATCCATC
Neo_Rev_probe	GCCACAGTCGATGAATCCAGAAA
cs_attH4X_R1	AAAACACAGCACGAGAACTTCGTGA
Neo 650 F	ACCGCTATCAGGACATAGCGTTGG
cs_attH4X_F1	GAGTGTTTTCCAACTTGGTTCCATT
cs_attH4X_F2	CCTGTCTTGCTAGGTTGGGAAGT
5.1 F	TCCCAGTTCAATTACAGCTCTTAAGCCCACCATGCAAATAGAGCTCTC
5.1R	TCGTATTAAGTACTCTAGCCTTAAGCTAGTAGAGGTCCTGTGCCTCG
7.1F	GCCCGGGAGATCTGCATGCCTGCAGGGCTCCGGTGCCCGTCAGTGGGC
7.2R	GGCTAGCGGATCCCCATGGCTGCAGCACACAAAAAACCAACACACAG
PGK_fwd_HR	CTGCAGCCATGGGGATCCGCTAGCGAATTCTACCGGGTAGGGGAGG
Puro_bpa rev_HR	CTTAATGAAATAAAGCAGGCTAGCCGACTCACTATAGGGCG
FLF8 348R	GGATACACCAACAGCATGAAG
FLF8 82R	TATAGTCCCATGACAGTTCCACTGC
Ch7 1175F	TTGATCTAATAGTGACAGTGGCGTG
Ch2 1282F	AGAATAATTGTGATGAGGTGCTGAG
ChX 1093F	TTGGGAGTCTGTCTCTATGTAGGTC

Ch7 440R	: TCCACTCAGAATCCCCATCCGAAGG
Ch2 440R	ACTCAGAAACCTCATTTGAAGGTC
ChX 831R	AGCATCAGCAGACTAAACGTTCTTG
hNANOG F	AGTCCCAAAGGCAAACAACCCACT TC
hNANOG R	TGCTGGAGGCTGAGGTATTTCTGTCTC
hOCT4 F	GACAGGGGGAGGGGAGGAGCTAGG
hOCT4 R	CTTCCCTCCAACCAGTTGCCCCAAAC
hSOX2 F	GGGAAATGGGAGGGGTGCAAAAGAGG
hSOX2 R	TTGCGTGAGTGTGGATGGGATTGGTG

Supplementary Table 2

List of genomic coordinates and locations of targeted attH4X in corresponding *LINE-1* in hESC clones using different primer pairs

**Clone #**	**Genomic co-ordinates**	**Genomic location**
Clone F1 and F9	Chr7:21928716-21928733	Intron of CDCA7L<br>(cell division cycle associated 7 like)
Clone B6	Chr2:178967015-178967032	Intron of CCDC141<br>(coiled-coil domain containing 141)
Clone B8	ChrX:32805376-32805393	Intron of dystrophin
Clone M27	Chr8: 81167096-81167177	Intron of HMGN5<br>(high mobility group nucleosome binding domain 5)
Clone T13	ChrX: 75815513-75815603	Intergenic
Clone T25	Chr5: 75249556-75249628	Intron of UPRT<br>(uracil phosphoribosyltransferase homolog)
